# Desmosomes in heart and skin: friends or foes?

**DOI:** 10.1186/s12967-024-05137-3

**Published:** 2024-04-16

**Authors:** Giuseppina Caiazzo, Rosa Redenta De Simone, Emanuele Monda, Ferdinando Barretta, Fabiana Uomo, Cristina Mazzaccara, Matteo Megna, Limongelli Giuseppe, Giulia Frisso

**Affiliations:** 1 Department of Advanced Biomedical Sciences, University of Naples Federico II, Napoli, Italy; 2 Department of Molecular Medicine and Medical Biotechnology, University of Naples Federico II, Naples, Italy; 3 CEINGE, Advanced Biotechnologies Franco Salvatore s.c.ar.l., Naples, Italy; 4 Inherited and Rare Cardiovascular Diseases, Department of Translational Medical Sciences, University of Campania Luigi Vanvitelli, Monaldi Hospital, Naples, Italy; 5 DAI Medicina di Laboratorio e Trasfusionale, AOU Federico II, Naples, Italy; 6 Section of Dermatology, Department of Clinical Medicine and Surgery, University of Naples Federico II, Naples, Italy

## Dear Editor,

Arrhythmogenic cardiomyopathy (ACM) is a rare and heritable heart-muscle disease that predisposes to ventricular arrhythmias potentially leading to sudden cardiac death (SCD), especially in young patients. Genetic mutations have been identified in about 50% of ACM patients. The most common mutations affect the genes encoding the desmosomal proteins: plakophilin-2 (*PKP2*), desmoglein-2 (*DSG2*), desmoplakin (*DSP*), and rarely, desmocollin-2 (*DSC*) and plakoglobin (*JUP*) [1]. Desmosomes are expressed in mechanical stressed tissue types, for instance epidermidis and cardiac muscle tissue, where they play a crucial role in maintaining cell adhesion and mechanical coordination. Therefore, mutations in desmosomal genes are associated with disorders involving heart (cardiomyopathies such as ACM and dilated cardiomyopathy), skin and hair (desmosomal genodermatoses). All desmosomal isoforms are expressed in the epidermidis, although in a layer-dependent manner; on the contrary, the expression is restricted to specific isoforms in the myocardium [2]. Despite the physiological role of desmosome structures is well established, both in epithelial tissue and cardiac muscle tissue, dermatological evaluations in ACM patients are not reported in the literature. Therefore, the aim of the current research is to investigate the presence of dermatological alterations in ACM patients carrying mutations in desmosomal genes. Patients (both sexes, age > 18 years) with a definite, borderline, or possible ACM diagnosis underwent molecular genetic testing, conducted at CEINGE_ Advanced Biotechnology Franco Salvatore of Naples, by analysing a panel of more than 100 genes associated with hereditary cardiomyopathies [3, 4]. 14 ACM patients carrying one or more variants in desmosomal genes agreed to carry out a dermatological check-up, at the Section of Dermatology of the University of Naples Federico II. The study was approved by the Ethics Committee of the University of Naples Federico II and conformed to the principles outlined in the Declaration of Helsinki. Each subject gave written informed consent before entering the study. The study population consisted of 7 females (mean age: 38 y) and 7 males (mean age: 27 y). Six patients were probands and were analyzed by NGS procedure; the remaining 8, belonging to three family groups, underwent family screening by searching for familial mutations. Table 1 reports genetic data of enrolled patients. As expected, most patients (79%) have mutations in the *PKP2* gene, followed by mutations in the *DSG2* (14%) and then in *DSP* genes (7%). Of note, *PKP2* and *DSG2* genes are expressed in the epidermal basal layer, *DSP* gene in all epidermal layer. All variants were classified according to the American College of Medical Genetics and Genomics (ACMG) guidelines, adapted to cardiomyopathies (maximal credible allele frequency for ACM was 0.000092) [4, 5]. Globally, we found 3 variants of unknown significance (VUS) and 2 pathogenetic variants (P) in the *PKP2* gene, one VUS in the *DSG2* gene, one likely pathogenic (LP) variant in *DES* gene and 1 variant classified as likely benign (LB) in the *DSP* gene. Even if this variant was classified as LB, the patient (AC-11) was a professional athlete, showing ECG abnormalities consistent with the ACM diagnosis and a positive family history for SCD; thus, we decided to include this patient in the analysis. Regarding dermatological phenotype, our study population was characterized by phototype II (*n* = 9, 64.3%), and phototype III (*n* = 5, 35.7%). Furthermore, 3 out of 14 patients (21.4%) showed a number of nevi < 10; 7 patients (50%) a number of nevi 10 < and < 50; and 4 (28.6%) > 50 nevi. Overall, 5 out of 14 participants (35.7%) reported a history of a previous dermatological disorder such as acne (*n* = 2, 14.2%), superficial fungal infections (*n* = 1, 7.1%), human papillomavirus infection (condyloma, *n* = 1, 7.1%) and basal cell carcinoma (*n* = 1, 7.1%). Six patients (42.8%) showed current cutaneous manifestations: plantar hyperkeratosis (*n* = 1, 7.1%), dyshidrotic eczema (*n* = 1, 7.1%), rosacea (*n* = 2, 14.2%), atypical nevi (*n* = 2, 14.2%). In addition, it has been evaluated the exposure to UV radiation during the life: never (*n* = 12, 85.7%), once or twice a year (*n* = 2, 14.3%). The sunscreen is usually used by 8 out of 14 patients (57.1%). Globally, the dermatological evaluation did not show any relevant features associated to genodermatoses. Consistent with these results, our study highlights that despite all the desmosomal isoforms are expressed in the epidermidis, the presence of genetic variants in desmosomal genes do not impair consistently the dermatological phenotype. Moreover, given that this study is in a preliminary stage, the small number of patients enrolled represents an important drawback. In conclusion, as a future prospective we plan to enlarge our study cohort and to further investigate whether the desmosomal genetic variants may affect the dermatological phenotype in ACM patients.

**Table 1:** Molecular results detected in 14 ACM patients who underwent dermatological analysis

Patients	ACM Diagnosis	Sex	Gene	HGVS coding (c.DNA)^@^	HGVS Protein Level^@^	Variant classification						
						Class of Variant	Reference SNP ID*	ClinVar$	HGMD§	ACMG Classification#	ACMG Supporting Criteria#	MAF (%) **
AC-1	possible	M	*PKP2*	c.764T > A	p.Leu255His	missense	NR	VUS	CM2011575	VUS	PM2, PP3, BP1	NR
AC-2	possible	F	*PKP2*	c.368G > A	p.Trp123Ter	nonsense	rs760576804	NR	CM118968	P	PVS1, PP5, PM2	NR
AC-3	definite	M	*PKP2*	c.368G > A	p.Trp123Ter	nonsense	rs760576804	NR	CM118968	P	PVS1, PP5, PM2	NR
			*PKP2*	c.764T > A	p.Leu255His	missense	NR	VUS	CM2011575	VUS	PM2, PP3, BP1	NR
AC-4	definite	M	*PKP2*	c.368G > A	p.Trp123Ter	nonsense	rs760576804	NR	CM118968	P	PVS1, PP5, PM2	NR
			*PKP2*	c.764T > A	p.Leu255His	missense	NR	VUS	CM2011575	VUS	PM2, PP3, BP1	NR
AC-5	possible	M	*DSG2*	c.1996G > C	p.Asp666His	missense	rs771623047	VUS	NR	VUS	PM2, PP3, BP1	0.0008
AC-6	borderline	M	*DSG2*	c.1996G > C	p.Asp666His	missense	rs771623047	VUS	NR	VUS	PM2, PP3, BP1	0.0008
AC-7	possible	F	*PKP2*	c.2134G > A	p.Gly712Arg	missense	rs200844640	NR	NR	VUS	BS2, BP1, PP3	0.001
AC-8	possible	M	*PKP2*	c.2134G > A	p.Gly712Arg	missense	rs200844640	NR	NR	VUS	BS2, BP1, PP3	0.001
AC-9	borderline	F	*PKP2*	c.2134G > A	p.Gly712Arg	missense	rs200844640	NR	NR	VUS	BS2, BP1, PP3	0.001
AC-10	borderline	M	*PKP2*	c.2134G > A	p.Gly712Arg	missense	rs200844640	NR	NR	VUS	BS2, BP1, PP3	0.001
AC-11	possible	F	*DSP*	c.4419 C > T	p.Ala1473Ala	synonymous	rs727504542	NR	NR	LB	BP6, BP4, BP7, PM2	0.006
AC-12	possible	F	*PKP2*	c.2013del	p.Lys672ArgfsTer12	frameshift	rs764817683	LP	CD061457	P	PVS1, PP5, PM2	0.0008
AC-13	definite	F	*PKP2*	c.1105 A > G	p.Arg369Gly	missense	NR	NR	NR	VUS	PM2, BP1	NR
AC-14	definite	F	*PKP2*	c.2013del	p.Lys672ArgfsTer12	frameshift	rs764817683	LP	NR	P	PVS1, PP5, PM2	0.0008

@: HGVS(https://hgvs-nomenclature.org); *:Reference SNP ID: NCBI SNPs Database (https://www.ncbi.nlm.nih.gov); $: Clinvar (https://www.ncbi.nlm.nih.gov); §: HGMD Professional 2023.4 (https://www.hgmd.cf.ac.uk/ac/index.php) ; #: ACMG Classification/Supporting Criteria (Richards S et al. Genet Med. 2015); **: Minor allele frequency (as in gnomAD: https://gnomad.broadinstitute.org); VUS = Variant of Uncertain Significance, NR = Not Reported, LP = Likely Pathogenic, P = Pathogenetic, LB = Likely Benign. Gene Transcripts: *PKP2* (NM_004572.4*)*, *DSG2* (NM_001943.5), *DSP* (NM_004415.4)

## Data Availability

All research data related to molecular analysis and clinical management of patients reported in the paper can be requested from the corresponding authors.
